# How AI Awareness Impacts Employee Adaptive Performance: A Moderated Mediation Model

**DOI:** 10.3390/bs16081294

**Published:** 2026-07-29

**Authors:** Hui Li, Yixuan Sun

**Affiliations:** School of Economics and Management, Nanjing Tech University, Nanjing 211816, China; lihui2012@njtech.edu.cn

**Keywords:** AI challenge awareness, AI hindrance awareness, job crafting, adaptive performance, mindfulness

## Abstract

Artificial intelligence (AI) has been widely adopted in workplaces and has significantly influenced how employees perform their work. AI awareness may influence employees’ adaptation to AI-enabled workplaces and changing job demands. Grounded in conservation of resources (COR) theory, our study tests the differential effects of AI challenge awareness and AI hindrance awareness on adaptive performance. Using three-wave survey data from 369 employees in manufacturing firms, we find that AI challenge awareness is positively related to adaptive performance, whereas AI hindrance awareness is negatively related to adaptive performance. Job crafting mediates the relationships between both forms of AI awareness and adaptive performance. Mindfulness positively moderates the relationship between job crafting and adaptive performance. Furthermore, mindfulness strengthens the positive indirect effect of AI challenge awareness on adaptive performance through job crafting while weakening the negative indirect effect of AI hindrance awareness through job crafting. The current study contributes to understanding how different forms of AI awareness impact adaptive performance. It also provides practical implications for how organizations can help employees adapt to AI-enabled workplaces.

## 1. Introduction

As artificial intelligence (AI) becomes increasingly integrated into the workplace, collaboration between humans and AI has grown more prevalent (Wilson & Daugherty, 2018). While AI brings benefits in cost reduction, precision, and efficiency, it also reshapes job demands and task structures. This transformation renders adaptive performance a critical competency in contemporary work contexts (Park & Park, 2019). However, rather than simply accepting AI-enabled workplace changes, employees form different perceptions of their implications. These individual perceptions are captured by the concept of AI awareness, which Brougham and Haar (2018) define as individuals’ perceptions of the implications of smart technology, artificial intelligence, robotics, and algorithms (STARA) for their career development and professional capabilities. This construct encompasses employees’ appraisals of both the challenges and hindrances associated with AI, as well as their expectations regarding future technological developments (Ding, 2021). Such awareness influences employees’ cognitive and behavioral responses to technological change and may facilitate successful adaptation in AI-enabled workplaces. Accordingly, it is critical to examine how AI awareness influences adaptive performance to help organizations effectively utilize AI in the workplace.

Adaptive performance is defined as behaviors through which employees adjust their knowledge and skills in response to changing environments and job requirements (Griffin et al., 2007). It also encompasses behaviors designed for managing, reacting to, and enabling change, thereby enhancing organizations’ capacity to navigate change (Jundt & Shoss, 2023). The deeper embedding of AI into work environments renders adaptive performance critical for change and innovation at the individual, group, and firm levels. When individuals fail to adapt to job demands, negative outcomes may ensue, including turnover intention (Kang et al., 2023), resistance to organizational change (Y. Wang & Han, 2020), and negative emotions (Park & Park, 2019), ultimately undermining both individual and organizational effectiveness. Prior research has shown that leadership behaviors, personality, and HRM practices are significantly linked to employees’ adaptive performance (Do et al., 2025; Huang et al., 2014). While AI empowers employees, it simultaneously increases performance pressure (Kang et al., 2023), posing substantial challenges to employees’ adaptive performance. Therefore, employees require adequate personal and job resources to cope with evolving work environments and job demands driven by AI. However, it remains largely underexplored whether and how AI awareness impacts employees’ adaptive performance, representing a notable void in prior research.

As a proactive form of work behavior, job crafting involves employees’ adjustments of their tasks, work relations, and cognitions so that work better fits their self-image, preferences, and values, thereby enhancing the meaningfulness of work (Wrzesniewski & Dutton, 2001). Job crafting has been shown to positively influence a range of work outcomes, including person–job fit, work engagement, creativity, and innovative behavior (Park & Park, 2023). COR theory (Hobfoll, 1989) posits that individuals are motivated to obtain, retain, and protect the resources they value. During organizational digital transformation, employees who engage in job crafting to cope with workplace changes can initiate a resource gain spiral, thereby enhancing their adaptive performance (Petrou et al., 2018). When individuals perceive AI as presenting challenge-related demands, they may invest resources by modifying their work tasks and work relations, with job crafting serving as a viable coping strategy (He et al., 2024). Conversely, when individuals perceive AI as a hindrance stressor that poses risks of job displacement or job insecurity, they may experience threats of resource loss, leading to negative cognitions and emotions. To avoid further resource depletion, withdrawal behaviors may emerge as a coping response (Kang et al., 2023). Therefore, job crafting may serve as an important mediator through which AI awareness influences adaptive performance.

How to enhance the positive effects of AI awareness and reduce its negative consequences has become a focal issue in understanding AI awareness’s influence on adaptive performance. Mindfulness is a positive individual trait characterized by a state in which individuals receptively attend to and accept ongoing internal and external stimuli while regulating their cognitions, emotions, and behavioral tendencies (Baer et al., 2004). Empirical research has shown that mindful employees who experience perceived work-related uncertainty and negative affective reactions are more likely to manage their behavior and attain adaptive performance (H. Liu et al., 2021). As a salient positive psychological state, mindfulness endows employees with sufficient psychological resources to cope with environmental changes and improve their well-being at work (Shahbaz & Parker, 2022). According to COR theory, psychological resources are critical in the process of resource gain or loss. Employees with abundant resources are better equipped to deal with stress or crises and are more likely to foster positive thinking (Hobfoll et al., 2018). Therefore, as a key psychological resource, mindfulness may help employees experience stress and adopt coping strategies differently, depending on its levels (Mubarak et al., 2022), which in turn influences the resource gain or loss process. This can lead to differential effects of job crafting on adaptive performance and help explain variations in the way AI awareness translates into adaptive performance via job crafting. Accordingly, mindfulness may serve as a critical boundary condition that moderates the associations linking AI awareness, job crafting, and employees’ adaptive performance.

In summary, drawing on COR theory, the current study introduces a moderated mediation framework to investigate the process through which AI awareness affects employees’ adaptive performance, addressing three core questions: whether AI awareness affects adaptive performance, how it does so through job crafting, and when it does so across different levels of mindfulness. The contributions of this study are twofold. First, it empirically demonstrates the divergent effects of AI challenge awareness and AI hindrance awareness on adaptive performance, enriching the scholarly understanding of AI awareness and extending empirical research on its consequences. Second, this study examines how AI awareness influences employee adaptive performance by identifying job crafting as a key explanatory mechanism and mindfulness as an important contextual factor, thereby offering theoretical guidance for employee performance management and work adaptation interventions in AI-enabled work contexts.

## 2. Literature Review and Theoretical Hypotheses

### 2.1. AI Awareness

With the rapid development and widespread application of AI, employees are increasingly exposed to and using AI systems in their work. AI not only enhances work efficiency, but also performs more complex tasks, fostering employees’ deep awareness of AI. AI awareness was first conceptualized by Brougham and Haar (2018) as the extent to which employees perceive AI as replacing existing jobs and threatening their career development. Their empirical findings demonstrated that AI awareness significantly and positively predicted employee depression, cynicism, and turnover intentions, while negatively predicting organizational commitment and career satisfaction. AI awareness is positively correlated with employees’ job insecurity, and employees who report higher levels of AI awareness show a greater tendency toward knowledge-hiding behavior (Tang et al., 2023). Xu et al. (2023) further confirmed, using a Chinese sample, that employees’ AI awareness is associated with emotional exhaustion, leading to negative psychological outcomes such as depression. AI awareness has been found to increase turnover intentions in the tourism and hospitality industry (J. J. Li et al., 2019). Since this definition is derived from job insecurity, such research has largely concentrated on the disruptive and threatening dimensions of AI, with outcomes predominantly reflecting the negative effects of AI awareness.

As a multilevel psychological construct, AI awareness can be conceptualized along multiple dimensions. Based on the transactional model of stress and cognitive appraisal (Searle & Auton, 2015), it can be categorized into challenge and hindrance dimensions, reflecting how employees perceive opportunities and obstacles associated with AI. Ding (2021) was among the first to propose this dual nature of AI awareness, arguing that employees not only view AI as a hindrance but also evaluate it as a challenge, which can enhance work engagement and organizational commitment, thereby stimulating innovation intentions. Subsequent studies have confirmed these positive effects. Liang et al. (2022) found that AI challenge awareness increases employees’ intrinsic motivation and promotes service innovation behaviors. Other research shows that AI challenge awareness can positively influence employee service performance through job crafting (He et al., 2024). H. Wang et al. (2022), using Chinese hotel employees as a sample, demonstrated that AI challenge awareness positively affects proactive learning and task crafting, ultimately enhancing employees’ creativity. Kong et al. (2024) found that AI challenge awareness strengthens employees’ career resilience, thereby improving informal learning behaviors. Taken together, these studies underscore the double-edged nature of AI awareness, highlighting the positive outcomes of individuals’ AI challenge awareness.

Given discrepancies in theoretical foundations and research perspectives, existing empirical studies have primarily adopted two approaches to operationalizing AI awareness. The first approach employs unidimensional scales. A typical unidimensional scale was developed based on job insecurity (Armstrong-Stassen, 2001) and consists of four items (Brougham & Haar, 2018), including statements such as “I think my job could be replaced by AI.” A number of previous studies have established the reliability and validity of this scale, which captures employees’ perceptions regarding their career prospects. The second approach employs two-dimensional scales. A representative two-dimensional scale was developed based on Searle and Auton’s challenge–hindrance stressor framework (2015) and comprises eight items (Ding, 2021), including challenge items such as “The job uncertainty generated from AI will help me to learn a lot” and hindrance items such as “The job uncertainty generated from AI will restrict my capability.” The two-dimensional conceptualization captures, on the one hand, employees’ concerns regarding AI-induced job replacement and, on the other hand, their cognitive appraisals of and planning for AI-enabled work contexts and career development.

Building on prior research, the present study categorizes AI awareness into challenge and hindrance dimensions based on the challenge–hindrance stressor framework and examines their differential effects on adaptive performance. AI challenge awareness is defined as employees’ perceptions of career and task opportunities arising from AI adoption, whereas AI hindrance awareness is defined as employees’ perceptions of obstacles that AI adoption poses to their job roles and career prospects. Overall, both types of AI awareness reflect employees’ perceptions of the impact of AI on work and careers, thereby shaping work-related resources and influencing adaptive performance.

### 2.2. AI Awareness and Employee Adaptive Performance

Adaptive performance reflects employees’ capacity to respond successfully to changing job requirements. In increasingly dynamic and uncertain work environments, organizations are expected to proactively recruit and develop employees with high levels of adaptability (Park & Park, 2019). Although scholars have yet to reach consensus on the conceptualization of adaptive performance, the construct consistently encompasses psychological activities and processes such as flexibility, resilience, emergency responsiveness, and coping with work-related stress. Pulakos et al. (2000) proposed an eight-dimensional taxonomy of adaptive performance, encompassing competencies such as managing crises, handling stress, and solving problems creatively. Building on this work, Griffin et al. (2007) conceptualized adaptive performance as behaviors directed toward adapting to, responding to, and supporting change at the task, team, and organizational levels. Existing research suggests that both individual and contextual factors, such as job skills, autonomy, motivation, social relationships, and perceived organizational and supervisory support, are associated with adaptive performance (Park & Park, 2019). In AI-enabled work contexts characterized by evolving job demands, adaptive performance has become increasingly important in employee management. For instance, continuous learning may help employees transfer AI-related knowledge and digital skills across changing task contexts (Billiot, 2023). Employees may also exhibit divergent cognitive and emotional responses to AI-induced changes in job demands, prompting different patterns of resource allocation and coping, which subsequently affect their adaptive performance.

Prior research has shown that AI challenge awareness helps employees develop capabilities for adjusting to changes in key tasks, improve the speed and quality of decision making under uncertainty, and creatively solve new problems arising from AI integration (He et al., 2024; Liang et al., 2022), thereby enhancing adaptability at the task level. At the same time, AI challenge awareness encourages employees to engage in proactive adaptive behaviors, including promotion-focused job crafting (Cheng et al., 2023), making them more likely to actively adjust their work methods and work relationships to accommodate new team collaboration requirements and role changes associated with AI adoption. Employees are also more inclined to establish positive human AI interactions and adapt to new team workflows and collaboration patterns, thereby enhancing adaptability at the team level. Furthermore, employees who are highly aware of AI challenges generally show stronger work engagement (Ding, 2021) and hold more open and positive attitudes toward AI driven organizational change. Research suggests that employees with positive attitudes toward organizational change are more likely to proactively adapt to new organizational practices and work arrangements (Oreg et al., 2011). Employees with high AI challenge awareness are also more willing to proactively acquire relevant information and skills to adapt to new organizational work systems, thereby enhancing adaptability at the organizational level. Overall, AI challenge awareness may promote adaptive performance by stimulating proactive adaptive behaviors across the task, team, and organizational levels.

In contrast, AI hindrance awareness causes employees to worry about job displacement and generates technological insecurity and anxiety (Brougham & Haar, 2018). The resulting depletion of cognitive resources undermines employees’ motivation to acquire new skills and weakens their confidence in managing uncertainty (Wu et al., 2024), leading to slower responses to changes in core tasks, lower decision-making quality, and difficulties in creatively solving new problems arising from AI adoption, thereby impairing adaptability at the task level. At the same time, AI hindrance awareness reduces employees’ acceptance of AI and encourages risk avoidance behaviors such as prevention focused job crafting (Mo et al., 2024). This conservative tendency lowers employees’ willingness to actively participate in team collaboration and human AI collaboration, making them more likely to maintain existing ways of working when facing new collaboration demands, thereby reducing adaptability at the team level. Furthermore, the job insecurity triggered by AI hindrance awareness continuously depletes employees’ psychological resources, fostering more negative and defensive attitudes toward AI-driven organizational change (Zhao et al., 2023) and reducing their willingness to adapt to new work systems, thereby weakening adaptability at the organizational level. Overall, AI hindrance awareness may inhibit adaptive performance by depleting employees’ resources and suppressing proactive adaptive behaviors across the task, team, and organizational levels.

COR theory posits that resources are important assets that help individuals achieve work goals, and that maintaining sufficient resources is critical for employees to accomplish their work objectives (Hobfoll et al., 2018). Prior research suggests that although AI awareness may heighten employees’ perceptions of job insecurity, it may also promote employees’ recovery and work engagement (S. Liu & Cheng, 2025), thereby influencing their adaptability. Employees with a high level of AI challenge awareness have a higher tendency to proactively invest resources in responding to changing job demands, thereby enhancing their adaptability. In contrast, AI hindrance awareness reflects employees’ perceptions of potential resource loss. The application of AI may reduce perceived organizational support for career development while increasing job mobility and the likelihood of career transitions (Zhang & Jin, 2023). Under such conditions, employees are more likely to conserve their limited psychological and energetic resources rather than invest them in adaptive behaviors, thereby reducing their adaptability. Overall, AI challenge awareness and AI hindrance awareness may be associated with employees’ adaptive performance through distinct resource gain and resource depletion pathways. Accordingly, the following hypotheses are proposed:

**H1a.**  *AI challenge awareness is positively related to employee adaptive performance.*

**H1b.**  *AI hindrance awareness is negatively related to employee adaptive performance.*

### 2.3. The Mediating Role of Job Crafting

Employees engage in job crafting as a bottom-up process, proactively modifying their tasks and relational boundaries, whether behaviorally or cognitively, in line with their own preferences and interests, so as to preserve a positive self-concept and derive value from work (Wrzesniewski & Dutton, 2001). More specifically, job crafting manifests as employees’ self-initiated adjustments to their work across task, relational, and cognitive dimensions, thereby aligning work more closely with the self and enhancing work meaningfulness. Job crafting typically encompasses three distinct dimensions: task, relational, and cognitive crafting (Slemp & Vella-Brodrick, 2014). Task crafting, a central component of job crafting, entails changing the quantity, range, or nature of work tasks. Relational crafting pertains to changes in the relational boundaries of work, involving adjustments in the quality or quantity of interactions with others. Cognitive crafting captures proactive shifts in cognitive strategies and work-related cognitions, thereby reshaping how work is perceived and evaluated. Although job crafting consists of three distinguishable dimensions, they are treated as complementary manifestations of an overarching proactive strategy, and therefore a unified construct approach is adopted. Consistent with this conceptualization, a substantial body of empirical research suggests that job crafting shows a positive relationship with various facets of job performance (Tims et al., 2012), including in-role, extra-role, and contextual performance.

The adoption of AI substantially reshapes employees’ traditional work characteristics and job resources. According to COR theory (Hobfoll, 1989), individuals experience psychological strain when they face threats of resource loss or when resource investments fail to yield expected returns. AI hindrance awareness reflects employees’ perceptions that organizational AI adoption may threaten their existing resources. Previously valuable skills and knowledge may lose their relevance in AI-enabled workplaces, thereby increasing the risk of resource loss. In addition, declines in organizational status and perceived organizational support may further deplete employees’ resources. The stronger employees’ AI hindrance awareness, the greater their perceived resource loss, which may contribute to psychological contract breach and emotional exhaustion (Bai et al., 2024). When individuals perceive AI adoption as a hindrance associated with potential resource loss, they have a greater propensity to exhibit defensive withdrawal behaviors by refraining from further resource investment and reducing job crafting. He et al. (2024) found that AI challenge awareness positively predicts job crafting, whereas AI hindrance awareness is not significantly related to job crafting. Kang et al. (2023) showed that service employees’ primary response to AI awareness is performance pressure, which in turn promotes job crafting. Cheng et al. (2023) demonstrated that employees may form challenge or hindrance appraisals of organizational AI adoption, which influence promotion-focused and prevention-focused job crafting, respectively.

Job crafting represents a proactive form of work behavior through which individuals take initiative to reconceptualize and reshape their work tasks, work methods, and interpersonal relationships. When individuals possess AI challenge awareness, they are more disposed to display such proactive behaviors. As a challenging job demand, AI challenge awareness can promote job crafting by enhancing employees’ motivation (H. Wang et al., 2022). Individuals who possess a higher level of AI challenge awareness exhibit stronger intrinsic motivation to cope with such demands through job crafting. In addition, AI challenge awareness may motivate employees to proactively modify their work. When employees believe that AI can facilitate goal attainment and career development, they are more intrinsically motivated to proactively seek ways to craft their jobs in response to emerging job demands (Cheng et al., 2023).

In contrast, when employees face hindrance job demands, they must invest additional effort and resources to cope with these demands, which may reduce their engagement in proactive behaviors. AI hindrance awareness reflects a hindrance appraisal and is likely to inhibit job crafting because coping with such hindrance demands requires substantial time and resources, while job crafting itself also consumes additional effort and resources (He et al., 2024). As job crafting involves employees’ self-initiated modifications to their job characteristics, engaging in such behaviors requires considerable determination, effort, and resources. AI hindrance awareness may further deplete employees’ limited resources, thereby reducing their involvement in job crafting. Concerns that AI may undermine employees’ job roles can reduce the resources available for job crafting. Existing evidence suggests that AI hindrance awareness is positively related to both physiological and psychological withdrawal behaviors (Nguyen & Nguyen, 2025). Accordingly, this study proposes the following hypotheses:

**H2a.**  *AI challenge awareness is positively related to job crafting.*

**H2b.**  *AI hindrance awareness is negatively related to job crafting.*

Research has shown that, from a resource perspective, job crafting contributes to greater work engagement and improved job performance (Tims et al., 2012). For example, seeking challenging job demands can enhance employees’ work adaptability (Petrou et al., 2018). Reflecting on work from a broader perspective can promote innovation-oriented cognitive processing, facilitate creative problem solving, and enhance informal learning (H. Wang et al., 2022). In addition, job crafting enables employees to demonstrate greater adaptability during organizational change (Vakola et al., 2023). Task crafting enables employees to integrate and optimize their job tasks while enhancing their work skills. By adjusting tasks, employees may generate new ideas and adaptive work behaviors that better align with the work environment and facilitate innovative problem solving (Bindl et al., 2019; Holman et al., 2024). Relational crafting may also enable employees to establish new collaborative relationships with AI, thereby enhancing their interpersonal competencies (Do et al., 2025). Meanwhile, relational crafting is associated with higher leader–member exchange quality and organizational identification, which are often accompanied by greater work engagement. Employees who value such commitment are more likely to achieve better person–job fit (Verelst et al., 2021). Even when work-related or interpersonal conflicts arise, employees may alleviate such conflicts by proactively cultivating positive relationships (Park & Park, 2019). In the context of digital transformation, employees can enhance their social interactions at work through communication with colleagues, thereby strengthening their interpersonal adaptability (Abhari et al., 2021). When employees adopt a positive attitude toward new job demands and invest greater effort in addressing task-related complexity, they are more likely to demonstrate higher adaptive performance. Accordingly, the hypothesis below is formulated:

**H3.**  *Job crafting is positively related to employee adaptive performance.*

COR theory posits that psychological resources boost individuals’ level of work engagement and their capacity to adjust to evolving work environments, thereby indirectly improving adaptability. Accordingly, employees with AI challenge awareness tend to perceive AI as a challenging job demand, which fosters job crafting and subsequently enhances adaptive performance. In contrast, employees with AI hindrance awareness perceive AI as a potential threat and negative stressor. As a result, they are less likely to proactively modify their work. This may further trigger withdrawal behaviors such as burnout, turnover intention, and disengagement (Teng et al., 2024), thereby inhibiting job crafting and ultimately impairing adaptive performance. Liang et al. (2022) argued that AI awareness influences innovative behavior through two distinct resource pathways by stimulating intrinsic motivation and increasing emotional exhaustion, respectively. Building on the theoretical arguments above, we hypothesize the following:

**H4a.**  *AI challenge awareness is positively related to adaptive performance through job crafting.*

**H4b.**  *AI hindrance awareness is negatively related to adaptive performance through job crafting.*

### 2.4. The Moderating Role of Mindfulness

Mindfulness refers to individuals’ purposeful attention to and conscious awareness of present-moment events and experiences, encompassing both state and trait dimensions (Brown & Ryan, 2003). By enhancing the breadth and quality of attention and alleviating stress and tension, mindfulness enables individuals to focus on present-moment information. Research has shown that employee mindfulness is strongly linked to job satisfaction, counterproductive work behavior, and task performance (Stuart-Edwards et al., 2023). In line with this, Dane and Brummel (2014) demonstrated the positive effect of mindfulness on job performance. Workplace mindfulness is primarily characterized by sustained attention, present-moment awareness, and non-judgmental acceptance (Baer et al., 2004; Dane & Brummel, 2014). Through the conscious regulation of emotions, cognitions, and behaviors, mindful individuals attend to internal and external stimuli with openness and non-judgment, thereby reflecting capacities for self-regulation and self-control (Good et al., 2016).

Workplace mindfulness enables employees to maintain focus on their work responsibilities and tasks while adjusting their behavioral responses to narrow the gap between ideal and actual work conditions. Mindfulness fosters continuous awareness of and attention to fluctuations in the work environment and job demands, thereby facilitating the active regulation of cognitions and emotional states and enhancing adaptability (Junça-Silva & Caetano, 2023). Through self-regulation, mindfulness influences adaptive performance by leveraging the interplay between personal and job resources (Zaw & Takahashi, 2022). Beyond task focus, mindfulness enhances interpersonal adjustment (Glomb et al., 2011) and also serves as a positive moderator in human–AI collaboration contexts.

Numerous studies have shown that mindfulness, particularly dispositional mindfulness, can moderate the relationship between stress coping strategies and performance. H. Liu et al. (2021) found that trait mindfulness acts as an important boundary condition of the indirect effects of evening cyber leisure. Montani et al. (2020) also found that as workload increases, employees with higher levels of mindfulness exhibit higher levels of work engagement. Notably, their engagement remains significantly higher than that of their low-mindfulness counterparts regardless of workload levels. Therefore, highly mindful employees possess greater initial resource pools to cultivate relational and social resources, maintain positive cognitions, and mobilize personal resources to cope with stress. This enables them to better adapt to evolving work relationships and tasks, thereby making job crafting more effective in improving adaptive performance. In contrast, employees with lower levels of mindfulness face a scarcity of personal resources and rely primarily on external job resources to sustain adaptability, which reduces the effectiveness of job crafting in promoting adaptive performance. Specifically, low-mindfulness employees are more prone to concerns regarding resource loss and may develop negative attitudes toward environmental changes, thereby making job crafting less effective in enhancing adaptive performance. Accordingly, the hypothesis below is formulated:

**H5.**  *Mindfulness positively moderates the relationship between job crafting and adaptive performance.*

Mindfulness promotes an accepting attitude toward internal and external stimuli and facilitates the processing of environmental information, enabling individuals to approach current work with greater clarity and positivity, develop more constructive work relationships, and adapt more effectively to organizational contexts, job demands, and collaboration with others. Prior research has identified individual characteristics, such as locus of control, core self-evaluations, emotional intelligence, and achievement motivation, as boundary conditions of the effect of AI awareness (Cheng et al., 2023; Z. Li et al., 2025; Nguyen & Nguyen, 2025; Tang et al., 2023). Wu et al. (2024) found that mindfulness alleviates the anxiety triggered by job insecurity arising from AI adoption, thereby mitigating its adverse impact on work- and life-related outcomes. Montani et al. (2020) found that mindfulness can buffer the detrimental consequences of increased workload and strengthen the positive effect of moderate workload on innovative work behavior via enhanced work engagement. Therefore, highly mindful employees are better equipped to regulate resources, remain focused on current work tasks, embrace challenging work tasks, and proactively reshape their work environment. They also show greater ability to deal with the stress associated with hindrance demands, improve their resource conditions through greater attention to internal and external cues, and mitigate the associated negative effects.

Integrating H4a, H4b, and H5, greater employee mindfulness is expected to strengthen the capacity of job crafting to promote adaptive performance in response to AI challenge awareness, while reducing its tendency to transmit the adverse influence of AI hindrance awareness. Conversely, lower employee mindfulness is likely to weaken the contribution of job crafting to adaptive performance under AI challenge awareness and intensify its transmission of the negative influence associated with AI hindrance awareness. Based on the above reasoning, the hypotheses are formulated as:

**H6a.**  *Mindfulness positively moderates the indirect effect of AI challenge awareness on adaptive performance through job crafting.*

**H6b.**  *Mindfulness negatively moderates the indirect effect of AI hindrance awareness on adaptive performance through job crafting.*

**Figure 1:**
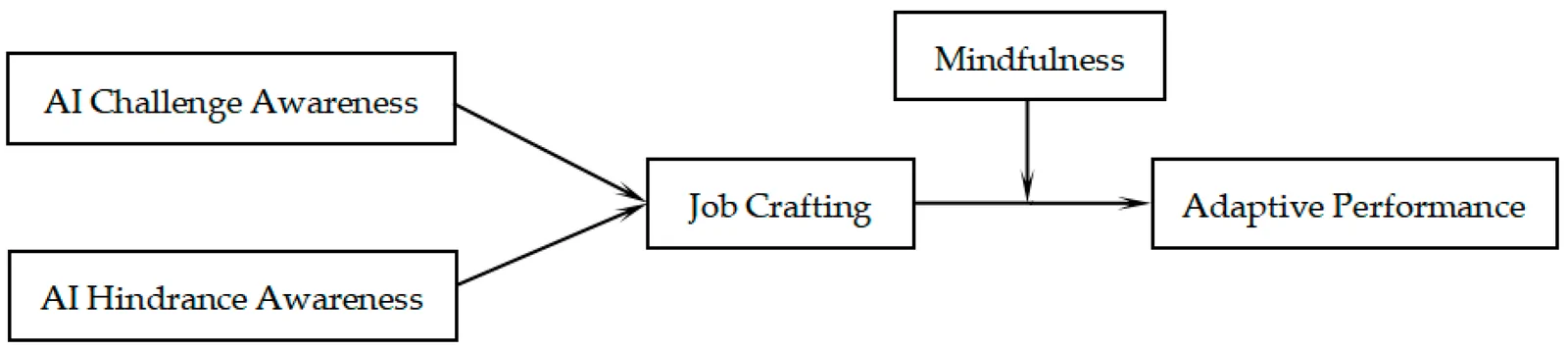
Theoretical research model.

In sum, Figure 1 illustrates the theoretical model of this study.

## 3. Methods

### 3.1. Sample and Procedure

This study recruited participants from ten intelligent manufacturing firms in the Suzhou Industrial Park. Data collection was facilitated by the park’s administrative committee and the human resources departments of the participating firms. The participating firms were all undergoing substantial digital and intelligent transformation initiatives and had widely adopted various AI-enabled systems into their daily operations. Employees across different functional areas, such as sales, operations, and R&D, interacted with AI systems, although the nature and intensity of AI exposure varied by job role. To facilitate matching across the three survey waves, questionnaires were coded using the four-digit suffix of each participant’s cell phone number. We assured participants that all responses would serve no purpose other than research and would be treated confidentially. The research team collected data across three waves spaced one month apart. At Wave 1 (T1), employees completed measures of demographic characteristics and AI awareness, yielding 687 responses. At Wave 2 (T2), employees completed measures of job crafting and mindfulness, yielding 526 responses. Finally, at Wave 3 (T3), employees completed measures of adaptive performance, yielding 385 responses. After matching responses across waves and excluding cases with missing data, the final usable sample consisted of 369 employees. In the final sample, 243 respondents were male (65.9%) and 126 were female (34.1%). Regarding age, 64 respondents (17.3%) were younger than 25 years, 128 (34.7%) were 26–30 years, 117 (31.7%) were 31–40 years, and 60 (16.3%) were aged 41 years or older. With respect to education, 59 respondents (16.0%) completed junior college or less, 219 (59.3%) completed a bachelor’s degree, and 91 (24.7%) completed a master’s degree or higher.

### 3.2. Measures

AI challenge awareness. To measure this construct, we administered Ding’s (2021) 4-item scale. A representative item is “The job uncertainty generated from AI will help me to learn a lot.” The Cronbach’s α was 0.859.

AI hindrance awareness. To measure this construct, we administered Ding’s (2021) 4-item scale. A representative item is “The job uncertainty generated from AI will restrict my capability.” The Cronbach’s α was 0.873.

Job crafting. For this variable, we employed the 15-item scale from Slemp and Vella-Brodrick (2014), which entails 3 subdimensions. The first was cognitive crafting (e.g., “Think about how your job gives your life purpose”), includes 5 items (Cronbach’s α was 0.831). The second was relational crafting (e.g., “Make an effort to get to know people well at work”), is composed of 5 items (α was 0.742). The third was task crafting (e.g., “Choose to take on additional tasks at work”), includes 5 items (α was 0.856). The Cronbach’s α of the second-order job crafting factor was 0.782.

Mindfulness. For this variable, we employed the 8-item scale from Brown and Ryan (2003). A representative item is “I rush through activities without being really attentive to them.” The Cronbach’s α was 0.814.

Adaptive performance. We measured this variable using Griffin et al.’s (2007) 9-item scale, which entails 3 subdimensions. The first, named individual task adaptivity (e.g., “Coped with changes to the way you have to do your core tasks”), includes 3 items (Cronbach’s α was 0.862). The second, named team member adaptivity (e.g., “Responded constructively to changes in the way your team works”), is composed of 3 items (α was 0.817). The third, named organization member adaptivity (e.g., “Coped with changes in the way the organization operates”), includes 5 items (α was 0.804). The Cronbach’s α of the second-order adaptive performance factor was 0.835.

Control variables. Employees’ demographic characteristics may exert direct effects on adaptive performance (Park & Park, 2019). Accordingly, we controlled for gender, age, and education, which were assessed at Wave 1 (T1).

### 3.3. Analysis Strategy

Before testing our hypothesized relationships, we used Mplus version 8.7 (Muthén & Muthén, 2017) to conduct a confirmatory factor analysis (CFA) using robust maximum likelihood estimation (MLR) to test the validity of our measurement model. Model fit was evaluated using the following indices and criteria: χ^2^/df < 3, CFI > 0.90, TLI > 0.90, IFI > 0.90, and RMSEA < 0.08. To test for mediating effects, bootstrapping with bias-corrected confidence estimates was conducted using 10,000 samples and 95 percent confidence intervals. To test the proposed moderated mediation model, we used PROCESS Model 14 (Hayes, 2013), with all continuous predictor variables mean-centered prior to analysis. Conditional indirect effects were examined to assess moderated mediation.

## 4. Results

### 4.1. Common Method Bias

Although the current study adopted a three-wave survey design, all variables were obtained from the same respondents, which may give rise to CMB. To examine this issue, Harman’s single-factor test was first conducted. The findings showed that five factors exhibited eigenvalues greater than 1, with the first factor explaining 34.159% of the total variance, below the recommended cutoff of 40%. These results suggest that CMB was unlikely to substantially affect the study variables (Podsakoff et al., 2003). Second, the unmeasured latent method factor technique was applied by incorporating a common method factor into the five-factor measurement model. The fit indices showed that CFI remained unchanged, TLI increased by 0.01, IFI remained unchanged, and RMSEA increased by 0.01, with all changes below the recommended criterion of 0.02, suggesting that the inclusion of the method factor did not meaningfully improve model fit (∆χ^2^ = 40.164, ∆df = 40). Overall, these findings suggest that CMB was unlikely to materially influence the study findings.

### 4.2. Discriminant Validity

This study included five key constructs: AI challenge awareness, AI hindrance awareness, job crafting, adaptive performance, and mindfulness. Discriminant validity was assessed through CFA in Mplus 8.7, with detailed results shown in Table 1. The five-factor model provided the best overall fit among all tested models (χ^2^(734) = 1099.032, CFI = 0.934, TLI = 0.922, IFI = 0.932, RMSEA = 0.069) and showed better fit than the competing models. These findings suggest that AI challenge awareness, AI hindrance awareness, job crafting, adaptive performance, and mindfulness represent distinct constructs, supporting discriminant validity among the variables.

### 4.3. Descriptive Statistics

Table 2 presents the descriptive statistics and correlations for the focal variables. Correlation analyses showed that AI challenge awareness was positively related to job crafting (r = 0.409, *p* < 0.01), whereas AI hindrance awareness was negatively related to job crafting (r = −0.284, *p* < 0.01). In addition, job crafting was positively associated with adaptive performance (r = 0.403, *p* < 0.01), and mindfulness was positively associated with adaptive performance (r = 0.251, *p* < 0.01). Overall, the correlations were generally consistent with the hypotheses.

### 4.4. Hypothesis Testing

Hypotheses were assessed using hierarchical regression analyses. Across all models, VIF values remained below the recommended cutoff of 10, suggesting that multicollinearity was unlikely to pose a serious issue. Table 3 summarizes the regression results.

First, Model 2 indicated that AI challenge awareness positively predicted job crafting (β = 0.408, *p* < 0.01), whereas AI hindrance awareness negatively predicted job crafting (β = −0.283, *p* < 0.01), supporting H2a and H2b. With adaptive performance entered as the dependent variable, Model 4 showed that AI challenge awareness positively predicted adaptive performance (β = 0.364, *p* < 0.01), whereas AI hindrance awareness negatively predicted adaptive performance (β = −0.187, *p* < 0.01), supporting H1a and H1b.

Second, Model 5 showed that job crafting positively predicted adaptive performance (β = 0.402, *p* < 0.05), supporting H3. In addition, the positive association of AI challenge awareness and the negative association of AI hindrance awareness with adaptive performance were both reduced but remained significant (β = 0.237, *p* < 0.01; β = −0.102, *p* < 0.01), suggesting that job crafting partially mediated both relationships and thereby supporting H4a and H4b. To further test the mediating effects, bootstrapping analyses with 5000 resamples were conducted. The 95% CIs for the indirect effects were estimated, and significance was determined based on whether the CIs excluded zero. Results showed that the indirect effect of AI challenge awareness on adaptive performance through job crafting was 0.163 (95% CI [0.079, 0.246]), further supporting H4a. Likewise, the indirect effect of AI hindrance awareness on adaptive performance through job crafting was −0.094 (95% CI [−0.154, −0.035]), further supporting H4b.

**Figure 2:**
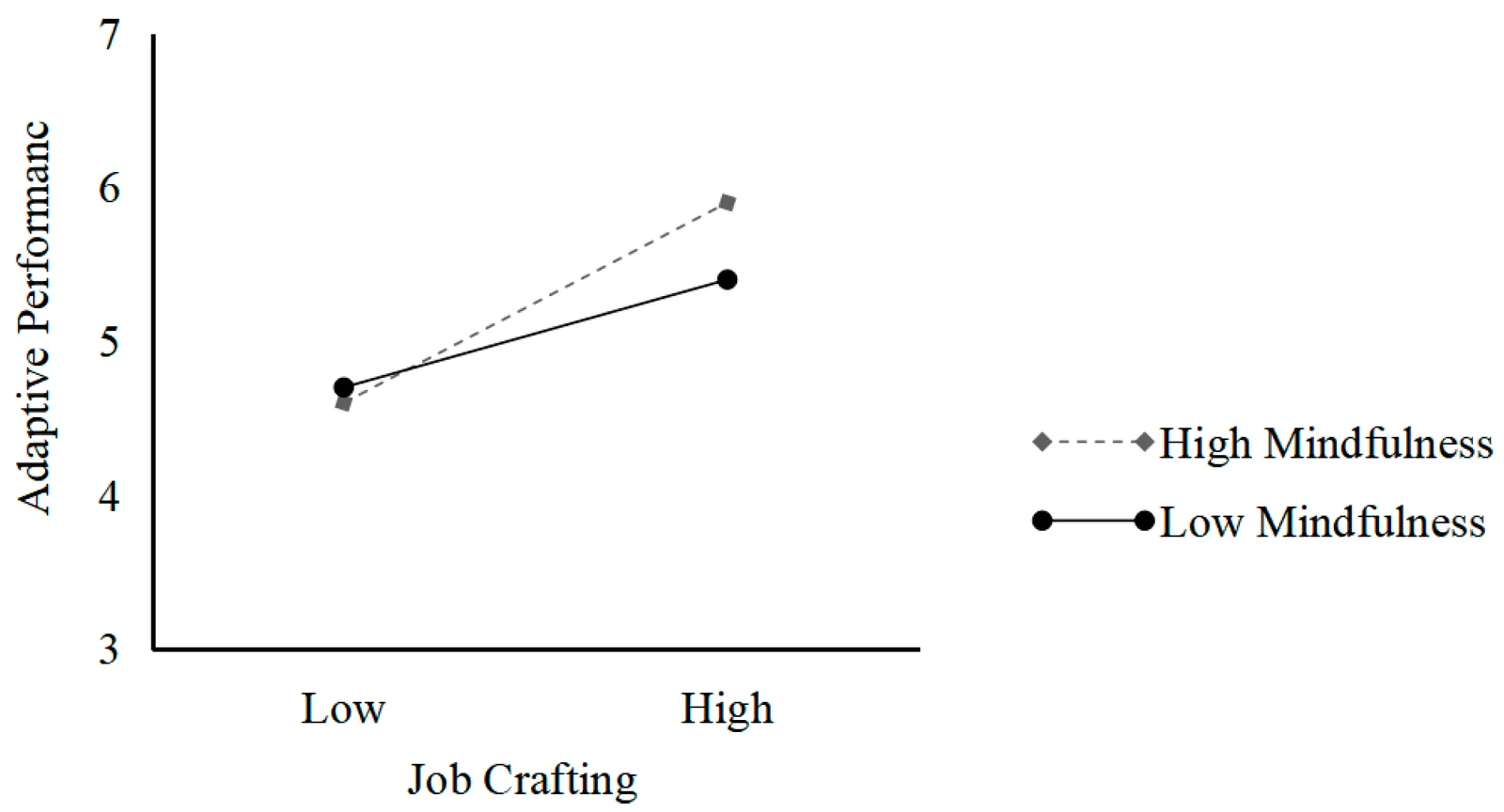
The moderating effect of mindfulness.

Third, after adding mindfulness and the interaction between mindfulness and job crafting, the interaction between mindfulness and job crafting was positively associated with adaptive performance in Model 6 (β = 0.276, *p* < 0.01), indicating that mindfulness strengthened the positive relationship between job crafting and adaptive performance and thereby supporting H5. Following standard procedures (Cohen et al., 2013), simple slope analyses were conducted at one standard deviation above and below the mean of mindfulness (M ± 1 SD) to illustrate the moderating effect. As shown in Figure 2, job crafting was positively associated with adaptive performance at both high levels of mindfulness (β_high_ = 0.387, *p* < 0.01) and low levels of mindfulness (β_low_ = 0.139, *p* < 0.01), although the relationship was weaker at low levels, further supporting H5.

Finally, to test the moderated mediation relationships, we applied the moderated path analysis approach (Edwards & Lambert, 2007) to estimatee two sets of effects at the high and low levels of the moderator. Specifically, the indirect effects of AI awareness on adaptive performance through job crafting were estimated at high (M + 1 SD) and low (M − 1 SD) levels of mindfulness, together with the corresponding 95% CIs for the differences between the conditional indirect effects. Table 4 summarizes the results. The indirect effect of AI challenge awareness on adaptive performance through job crafting remained significant at both high and low levels of mindfulness. Moreover, the difference between the two conditional indirect effects was 0.092 (95% CI [0.052, 0.132]), suggesting that mindfulness positively moderated the indirect effect and thereby supporting H6a. Similarly, the negative indirect effect of AI hindrance awareness on adaptive performance through job crafting remained significant across both levels of mindfulness. The difference between the two conditional indirect effects was 0.079 (95% CI [0.048, 0.114]), showing that mindfulness negatively moderated the indirect effect and thereby supporting H6b.

## 5. Conclusions

Drawing on COR theory, this study constructed a theoretical framework linking AI awareness, job crafting, and adaptive performance and empirically examined the mechanisms and boundary conditions through which employees’ AI awareness, under different appraisals, influences adaptive performance. Results revealed that AI challenge awareness significantly and positively predicted adaptive performance, whereas AI hindrance awareness significantly and negatively predicted adaptive performance, and that job crafting partially mediated both relationships. Furthermore, mindfulness strengthened the positive effect of job crafting on adaptive performance. It also amplified the positive indirect effect of AI challenge awareness on adaptive performance through job crafting, while weakened the negative indirect effect of AI hindrance awareness through job crafting. Specifically, when mindfulness was high, AI challenge awareness exhibited stronger positive associations with job crafting and adaptive performance (via job crafting), whereas the adverse indirect influence of AI hindrance awareness on adaptive performance was weaker. Conversely, at low levels of mindfulness, these patterns were reversed. Therefore, these results point to the conclusion that the effects of AI awareness on job crafting and adaptive performance depend on whether individuals appraise AI adoption as resource gains or resource losses, as well as on their level of mindfulness. This investigation extends previous work on AI awareness and provides practical implications for managing and facilitating employee adaptation.

### 5.1. Theoretical Implications

First, our article advances existing work on AI awareness and adaptive performance. AI awareness enables employees to assess the impact of AI on their work and proactively adopt coping strategies to achieve adaptation in AI-enabled work environments. The article employed a challenge–hindrance framework to measure AI awareness, offering a more thorough investigation of its effects and extending previous work that focused solely on job insecurity arising from job displacement concerns. Moreover, while prior research has examined AI awareness in relation to career development and employee growth (Brougham & Haar, 2018), the primary task when AI is applied in the workplace is employee adaptation. Only on this basis can employees exhibit enhanced in-role and extra-role performance, such as organizational citizenship behavior and career advancement. Additionally, this study enriches understanding of how employees flexibly adjust and integrate their resources in dynamic work environments (Petrou et al., 2018), achieving work adaptation through effective self-management and responses to external challenges.

Second, our results indicate that job crafting transmits the effect of AI awareness to adaptive performance. Employees adopt this proactive, individual-level coping mechanism when responding to AI. He et al. (2024) found that AI challenge awareness positively influenced job crafting, and the present study corroborates this finding while further revealing a negative effect of AI hindrance awareness on job crafting. Moreover, by integrating COR theory (Hobfoll, 1989; Hobfoll et al., 2018) and self-regulation theory (Good et al., 2016) with the core concepts of job crafting theory (Wrzesniewski & Dutton, 2001), the present research advances existing work on the mechanisms underlying the effects of AI awareness from a job crafting perspective. Based on survey data from employees in manufacturing firms, this study incorporated the two facets of AI awareness and empirically tested employees’ coping strategies and their behavioral effects in response to AI challenge and hindrance awareness within digital work environments. Consequently, this study enhances knowledge of the pathways through which AI awareness influences adaptive performance, tests the resource gain spiral posited by COR theory (Hobfoll, 1989; Hobfoll et al., 2018), and enriches research on how job crafting transmits these effects.

Third, this study identifies important boundary conditions of AI awareness effects. Prior studies have investigated contextual factors, such as core self-evaluations and perceived organizational support, in the linkage between AI awareness and work outcomes (He et al., 2024; Tang et al., 2023). Distinct from these studies, the present research positions mindfulness as a critical psychological resource that enhances adaptive strategies for acquiring job resources, thereby offering new insights into how individual psychological traits interact with AI awareness to influence adaptive performance. Additionally, this study responds to Stuart-Edwards et al.’s (2023) call to explore the association between mindfulness and adaptive performance in challenging work domains such as digitalization and AI. Based on data from Chinese manufacturing firms, our article confirms the boundary role of mindfulness in the connections among AI awareness, job crafting, and adaptive performance, enhancing our understanding of mindfulness’s adaptive role in uncertain environments and contributing to deeper insights into the psychological dynamics between AI technology application and employee responses.

### 5.2. Practical Implications

First, our study provides practical implications for managing employee AI awareness and enhancing adaptive performance in organizations. Organizations should understand employees’ attitudes toward AI, including both positive and negative evaluations, and examine their cognitive and emotional responses to AI adoption. On this basis, targeted interventions combining communication, participation, and feedback mechanisms can be developed to monitor heterogeneity in AI awareness and its evolution over time (Gemmano et al., 2026). Managers have a key part to play in supporting adaptation by helping employees recognize skill upgrading as essential for working with AI technologies. Through communication, training, and psychological support, employees can develop more positive interpretations of AI, including the recognition that AI can enhance work capabilities, foster innovation, and enhance work meaningfulness. Organizations should also invest in AI-related training to align employee competencies with technological requirements, thereby improving adaptive performance. Finally, cultivating a culture of human–AI collaboration can further facilitate employees’ adjustment to AI-enabled work environments.

Second, job crafting is a proactive coping strategy that allows employees to autonomously alter their work characteristics in AI-enabled workplace contexts. Organizations should enhance employee work autonomy, thereby creating conditions that enable job crafting, and encourage individuals to adjust their task, relational, and cognitive boundaries based on job characteristics and AI interaction patterns. Managers can also use performance appraisal feedback to guide employees in making targeted work adjustments. Organizations should further support job crafting by facilitating cognitive reframing of AI-enabled work environments, strengthening AI-related skill development, and providing training to enhance employees’ AI knowledge. In addition, organizations can enable employees to adjust their own task, relational, and cognitive crafting in reaction to AI and individual attitudes. Such support may be implemented through interventions such as work analysis, planning, and reflection processes (Demerouti et al., 2019).

Third, the widespread adoption of AI in workplaces exposes employees to significant technological disruptions, leading to emotional reactions such as anxiety and insecurity, as well as reduced well-being and adaptation difficulties, thereby posing challenges for organizational human resource management. Therefore, organizations should recognize the important role of mindfulness in facilitating adaptive coping with AI adoption and integrate mindfulness into employee selection, placement, training, and development. Organizations may select employees with higher levels of mindfulness during recruitment, thereby establishing a foundation for adaptation in AI contexts. They can also incorporate mindfulness training into internal development systems, drawing on practices from firms such as Intel and Google. Through such training, employees can better manage attention, alleviate AI-induced stress and insecurity, and enhance work adaptability (Marsh et al., 2024). Organizations may further cultivate a mindful organizational climate by providing time for mindfulness practices and dedicated spaces, thereby supporting employee adaptability and sustainable organizational development.

### 5.3. Limitations and Future Research

Although this research makes notable contributions, it also presents limitations that warrant further investigation.

First, the sample came mainly from employees in selected enterprises within an industrial park, which could constrain the generalizability of these findings. Subsequent work might employ experience sampling methods, scenario-based experiments, or other related methodological approaches to further validate these results.

Second, all core variables were measured using employee self-reports. Although the multi-wave design reduced common method bias procedurally, the single-source data may still be influenced by social desirability or consistency motives, inflating the relationships among variables. Future research could incorporate multisource data, such as supervisor ratings, to address this limitation.

Third, this study tested hypotheses using hierarchical regression rather than structural equation modeling (SEM). Consequently, we were unable to simultaneously estimate all path relationships while controlling for measurement errors, nor could we provide overall model fit indices. Future research could employ latent variable SEM to cross-validate our hypothesized model.

Fourth, while this study examined mindfulness as a moderator, future research may explore additional boundary conditions such as dialectical thinking and paradoxical leadership.

Fifth, job crafting and adaptive performance may reflect a reciprocal process, whereby adaptive performance may also serve as a mediator of the linkage between AI awareness and job crafting. Employees with stronger adaptive capabilities may show a greater tendency to perform job crafting when reacting to AI awareness. Subsequent studies could use longitudinal designs to examine these dynamic relationships with greater rigor.

Sixth, drawing on COR theory, the present study primarily focused on the logic of resource loss expectation, yet it did not examine another resource-depletion pathway, AI fatigue (Lau et al., 2026). AI fatigue may deplete employees’ cognitive and emotional resources required for job crafting, thereby partially explaining why AI hindrance awareness undermines job crafting. Since we did not incorporate a measure of AI fatigue, we are unable to determine whether this pathway operates. Future research could introduce the AI Fatigue Scale to further test its mediating role in the relationship between AI hindrance awareness and job crafting.

Finally, given the important role of organizations and managers in employee adaptation to AI-driven change, subsequent studies might include variables like coaching leadership and high-involvement work systems into theoretical models to examine their antecedent or boundary effects and extend the analysis to team- and organizational-level contexts.

## Figures and Tables

**Table 1:** Results of confirmatory factor analysis.

Model	χ^2^	df	χ^2^/df	∆χ^2^ (∆df)	CFI	TLI	IFI	RMSEA
Five-factor model	1099.032	734	1.497	—	0.934	0.922	0.932	0.069
Four-factor model	1992.241	730	2.729	893.209(4) ***	0.916	0.889	0.914	0.083
Three-factor model	2964.078	727	4.077	971.837(3) ***	0.857	0.835	0.856	0.105
Two-factor model	4026.165	725	5.553	1062.087(2) ***	0.729	0.703	0.726	0.128
Single-factor model	5352.858	724	7.393	1326.693(1) ***	0.594	0.528	0.583	0.174

Note: *** *p* < 0.001. Five-factor model: CAIA, HAIA, JC, AP, MF; Four-factor model: CAIA + HAIA, JC, AP, MF; Three-factor model: CAIA + HAIA, MF, JC + AP; Two-factor model: CAIA + HAIA, JC + AP + MF; Single-factor model: CAIA + HAIA + JC + AP + MF. CAIA = AI challenge awareness; HAIA = AI hindrance awareness; JC = job crafting; AP = adaptive performance; MF = mindfulness.

**Table 2:** Descriptive statistics and correlations.

Variable	M	SD	1	2	3	4	5	6	7	8
1. Gender	0.659	0.474	—							
2. Age	29.327	6.138	0.059	—						
3. Education	1.341	0.872	0.032	0.103	—					
4. AI challenge awareness	5.631	0.796	0.081	0.032	0.113	**0.605**				
5. AI hindrance awareness	5.107	0.835	0.079	0.054	0.072	−0.157 *	**0.593**			
6. Job crafting	5.214	0.851	0.057	0.081	0.048	0.409 **	−0.284 **	**0.579**		
7. Mindfulness	5.428	0.937	0.064	0.074	0.059	0.145 **	−0.207 **	0.193 *	**0.628**	
8. Adaptive performance	5.385	0.753	0.035	0.057	0.027	0.364 **	−0.186 **	0.403 **	0.251 *	**0.641**

Note: * *p* < 0.05, ** *p* < 0.01. Boldface values on the diagonal represent AVE (average variance extracted) values.

**Table 3:** Results of hierarchical regression analysis.

Variable	Job Crafting		Adaptive Performance			
	M 1	M 2	M 3	M 4	M 5	M 6
**Control variables**						
Gender	0.058	0.057	0.035	0.034	0.034	0.035
Age	0.082	0.081	0.059	0.058	0.058	0.059
Education	0.047	0.048	0.027	0.027	0.026	0.026
**independent variable**						
AI challenge awareness		0.408 **		0.364 **	0.237 **	0.223 **
AI hindrance awareness		−0.283 **		−0.187 **	−0.102 **	−0.105 **
**Mediator**						
Job crafting					0.402 *	0.314 *
**Moderator**						
Mindfulness						0.252 *
**Interaction term**						
Job crafting × Mindfulness						0.276 **
R^2^	0.035	0.263	0.041	0.249	0.442	0.645
ΔR^2^	0.035	0.228	0.041	0.208	0.193	0.203
F	1.298	5.667 **	1.596	6.985 **	9.465 **	14.234 **

Note: * *p* < 0.05, ** *p* < 0.01.

**Table 4:** Conditional indirect effects of AI awareness on adaptive performance.

Independent Variable	Moderator	Conditional Indirect Effects		
		β	SE	95% CI
AI challenge awareness	High mindfulness	0.224 **	0.042	[0.115, 0.332]
	Low mindfulness	0.112 *	0.028	[0.084, 0.151]
	Difference	0.092 **	0.035	[0.052, 0.132]
AI hindrance awareness	High mindfulness	−0.047 **	0.034	[−0.069, −0.023]
	Low mindfulness	−0.126 *	0.025	[−0.149, −0.103]
	Difference	0.079 *	0.029	[0.048, 0.114]

Note: * *p* < 0.05, ** *p* < 0.01.

## Data Availability

The data presented in this study are available on request from the corresponding author due to privacy and ethical restrictions.

## Abbreviations

The following abbreviations are used in this manuscript:

AI	Artificial Intelligence
COR	Conservation of Resources
CMB	Common Method Bias
CFA	Confirmatory Factor Analysis
VIF	Variance Inflation Factor
CI	Confidence Interval
AVE	Average Variance Extracted
CFI	Comparative Fit Index
TLI	Tucker–Lewis Index
IFI	Incremental Fit Index
RMSEA	Root Mean Square Error of Approximation
SD	Standard Deviation
SEM	Structural Equation Modeling
